# Label-Free Detection of Salivary Pepsin Using Gold Nanoparticle/Polypyrrole Nanocoral Modified Screen-Printed Electrode

**DOI:** 10.3390/s18061685

**Published:** 2018-05-24

**Authors:** Doyeon Lee, Young Ju Lee, Young-Gyu Eun, Gi-Ja Lee

**Affiliations:** 1 Department of Medical Engineering, Graduate School, Kyung Hee University, Seoul 130-701, Korea; lghee1201@naver.com; 2 Department of Biomedical Engineering, College of Medicine, Kyung Hee University, Seoul 130-701, Korea; younglee@khu.ac.kr; 3 Department of Otolaryngology-Head and Neck Surgery, Kyung Hee University Medical Center, Seoul 130-702, Korea; ygeun@hanmail.net

**Keywords:** pepsin, saliva, laryngopharyngeal reflux, polypyrrole nanocorals, electrochemical immunosensor

## Abstract

Detection of salivary pepsin has been given attention as a new diagnostic tool for laryngopharyngeal reflux (LPR) disease, because saliva collection is non-invasive and relatively comfortable. In this study, we prepared polypyrrole nanocorals (PPNCs) on a screen-printed carbon electrode (SPCE) by a soft template synthesis method, using β-naphthalenesulfonic acid (NSA) (for short, PPNCs/SPCE). Gold nanoparticles (GNPs) were then decorated on PPNCs/SPCE by electrodeposition (for short, GNP/PPNCs/SPCE). To construct the immunosensor, pepsin antibody was immobilized on GNP/PPNCs/SPCE. Next, citric acid was applied to prevent non-specific binding and change the electrode surface charge before pepsin incubation. Electrochemical stepwise characterization was performed using cyclic voltammetry, and immunosensor response toward different pepsin concentrations was measured by differential pulsed voltammetry. As a result, our electrochemical immunosensor showed a sensitive detection performance toward pepsin with a linear range from 6.25 to 100 ng/mL and high specificity toward pepsin, as well as a low limit of detection of 2.2 ng/mL. Finally, we quantified the pepsin levels in saliva samples of LPR patients (*n* = 2), showing that the results were concordant with those of a conventional ELISA method. Therefore, we expect that this electrochemical immunosensor could be helpful for preliminarily diagnosing LPR through the detection of pepsin in saliva.

## 1. Introduction

Laryngopharyngeal reflux (LPR) disease refers to the backflow of gastric juice containing acid and pepsin into the laryngopharynx, causing irritation of the vocal folds and the surrounding mucous membrane [1]. Although 24-h double-probe pH monitoring has been considered as a gold standard diagnostic test for LPR, it is not widely used in all suspected cases of LPR because of its invasiveness and cost [2,3]. Therefore, it is necessary to develop a reliable diagnostic method that is non-invasive, cost-effective, and more specific for LPR.

Pepsin is an enzyme that is produced by chief cells of the stomach and digests protein. Studies have reported that, damage caused by LPR is attributed to not only acid reflux, but also non-acid reflux, such as pepsin [4,5]. Though it is known that laryngeal mucosa is resistant to acidic material above pH 4, the presence of pepsin can damage laryngeal tissue, even in mild acidic or alkaline environments [6,7]. Therefore, pepsin has received the attention of clinicians as a potential biomarker for LPR diagnosis. Moreover, detection of pepsin in saliva can be a new diagnostic tool for LPR because of its non-invasive, easy, and comfortable method of sample collection [8,9,10]. However, the concentration of salivary pepsin is 1000 times lower than that of gastric juice [11], and many other interfering proteins are present in the saliva [12]. In addition, saliva for the detection of pepsin should be collected using an acidic solution, such as citric acid (pH 2.5), to preserve the enzymatic activity. Therefore, it is necessary to develop a more sensitive and selective detection method for salivary pepsin in acidic conditions.

Currently accepted methods for the detection of pepsin include enzyme-linked immunosorbent assay (ELISA) [9], enzymatic assay with a fluorescent substrate [13] and Western blot analysis [14,15]. However, these methods have limitations of their use because of long time for analysis and complex experimental process. Recently, Peptest^TM^ lateral flow device (LFD) (RD Biomed Ltd., Hull, UK) has been developed as a convenient, office-based, noninvasive and quick technique for pepsin determination with the use [16]. Although LFDs feature operational simplicity and good selectivity derived from antigen-antibody immunoreactions, their low detection sensitivity is a remaining issue. 

Conducting polymer-based electrochemical biosensors have received great attention, because they are easy and cost-effective to fabricate, and also show superior sensing performances, including high sensitivity, selectivity, and rapid response time [17]. Among the various conducting polymers, polypyrrole (PPy) is the most commonly used component in immunosensors, owing to its high biocompatibility, efficient polymerization in aqueous solutions at neutral pH, capability to protect electrodes from interfering materials, and applicability in biosensor and immunosensor applications [18,19]. In particular, nanostructured conducting PPy can provide excellent sensitivity via enhanced interactions between conducting polymers and analytes, as a result of a high surface-area-to-volume ratio [20]. Conducting polymer nanostructures, such as nanowires, nanospheres, and nanoribbons, have been synthesized with template or self-assembly methods [21]. Hard template synthesis methods require appropriate porous materials as a template, which are removed after synthesis of the nanomaterials. On the other hand, self-assembly methods often utilize soft templates, which contain a long-range ordered structure self-assembled from certain surfactants or block copolymers [22]. The main advantage of soft template synthesis methods is that they are easily removed after synthesis of the nanostructures of the resulting polymers. In addition, gold nanoparticles (GNPs) are widely used in this field, due to their capacity for significantly enhancing transduction signals of a sensing system [23]. GNPs possess unique properties, such as rich surface chemistry, low toxicity, high electron density, and strong optical absorption [24]. Because this biologically inert material does not affect molecular structure or activity, GNPs can be easily functionalized with biological macromolecules to allow for detection using a variety of optical and electrochemical transduction mechanisms [7]. Another beneficial characteristic of GNPs is the large surface area of their structure relative to their volume, which can be used as a means of functionalizing larger quantities of proteins compared with using planar electrodes or even nanomaterials with different geometries [23]. Therefore, it is expected that GNP-decorated conducting polymer nanostructures can not only easily immobilize antibodies, but also enhance the electrical properties of the nanoparticles, thereby improving immunosensor performance.

**Figure 1:**
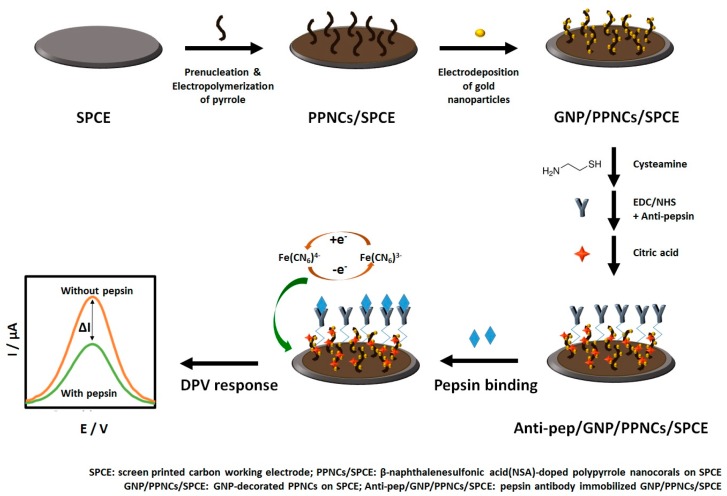
Schematic illustration of the fabrication process of the electrochemical immunosensor based on GNP/PPNCs/SPCE for pepsin detection.

In this study, we prepared a facile and sensitive immunosensor by using GNP-decorated PPy nanocorals (PPNCs) on a screen-printed carbon electrode (SPCE) for the detection of pepsin in saliva. PPNCs were electropolymerized on an SPCE using β-naphthalenesulfonic acid (NSA) as a surfactant dopant (for short, PPNCs/SPCE). GNPs were then deposited on PPNCs (for short, GNP/PPNCs/SPCE). For selective pepsin detection, pepsin antibody (anti-pep) was immobilized on GNPs. Each step of immunosensor fabrication was confirmed by cyclic voltammetry (CV). The differential pulsed voltammetric (DPV) response of ferricyanide, used as a redox probe in the label-free approach, was measured to monitor the affinity reactions between antibody and antigen onto the immunosensor. For real sample applications, the as-prepared immunosensor was preliminarily evaluated by determining the concentration of pepsin in the saliva of LPR patients (*n* = 2). The electrochemical immunosensing system for the detection of pepsin in saliva is schematized in Figure 1.

## 2. Materials and Methods

### 2.1. Materials and Apparatus

Pyrrole monomer (reagent grade, 98%), hydrogen tetrachloroaurate (III) trihydrate (HAuCl_4_·3H_2_O), sulfuric acid (H_2_SO_4_), potassium chloride (KCl), 2-naphthalenesulfonic acid (NSA), NHS, EDC, cysteamine (CA), potassium hexacyanoferrate (III) (K_3_Fe(CN)_6_), potassium hexacyanoferrate (II) trihydrate (K_4_Fe(CN)_6_·3H_2_O), lysozyme human, α-amylase from human saliva, bovine serum albumin (BSA), and human serum albumin were purchased from Sigma-Aldrich (St. Louis, MO, USA). Citric acid monohydrate was obtained from Junsei Chemical Co. Ltd. (Chuo-ku, Tokyo, Japan). Polyclonal pepsin antibody (pAA165Hu01) and pepsin (CPA632Hu21) were purchased from Cloud-Clone Crop (Katy, TX, USA).

All electrochemical experiments, including CV and differential pulse voltammetry, were carried out with a Compactstat (Ivium Technology, Eindhoven, The Netherland). The screen-printed carbon electrode (SPCE, C110) containing a carbon working electrode (4 mm in diameter) and screen-printed gold electrode (SPGE, C220AT) containing a gold working electrode (4 mm in diameter) were purchased from DropSens (DRP-C110, Llanera, Asturias, Spain). The electrodes consisted of a carbon counter electrode and a silver pseudo-reference electrode. Other solvents and chemicals were analytical reagent grade and were used as received. All aqueous solutions were prepared using distilled water of 18.2 MΩ·cm resistivity. All experiments were carried out at room temperature. The morphologies of the working electrode surfaces were characterized using a field emission scanning electron microscope (FE-SEM; S-4700, Hitachi, Tokyo, Japan).

### 2.2. Fabrication of GNP/PPNCs/SPCE

Prior to modification, SPCE was activated by CV scanning in 1 M H_2_SO_4_ at a scan rate of 100 mV·s^−1^ with a potential range of −0.5 to 1.0 V (vs. silver pseudo-reference electrode) for five cycles. First, a pre-nucleation film for PPNCs on the activated SPCE was prepared potentiostatically at 0.8 V (vs. silver pseudo-reference electrode) for 20 s in 0.2 M KCl solution as the electrolyte containing 0.1 M pyrrole, using a previously reported method [19,21,22]. After washing the films in de-ionized water, electrochemical polymerization was performed potentiostatically at 0.6 V (vs. silver pseudo-reference electrode) for 120 s in a phosphate buffer (PB, 0.5 M, pH 6.8) solution containing 0.2 M pyrrole and 0.01 M NSA to prepare the NSA-doped PPNCs on pre-nucleated SPCEs. Finally, electrodeposition of GNPs on PPNCs/SPCE was carried out using CV over ten cycles at a potential range from −1.0 to 0.2 V (vs. silver pseudo-reference electrode) and at a scan rate of 50 mV·s^−1^ in a 0.1 M KCl aqueous solution containing 0.5 mM HAuCl_4_·3H_2_O. The GNP/PPNCs/SPCE was washed with de-ionized water and dried at room temperature.

### 2.3. Preparation of the GNP/PPNCs/SPCE-Based Immunosensors

The GNP/PPNCs/SPCE was incubated in a 1 mM CA aqueous solution for 2 h at room temperature in darkness to allow the assembly of CA on the surface of GNPs. Subsequently, the electrode was washed with de-ionized water for 2 min. The CA-modified electrode was then incubated in an anti-pepsin (1 μg/mL) and EDC (2 mM)/NHS (5 mM) solution for 1 h. After the anti-pepsin immobilization step, the electrode was washed with phosphate buffer saline (PBS, pH 7.4). To block potential remaining active sites of GNPs against non-specific binding, the electrode was incubated for 30 min in 0.1 M citric acid (pH 2.5), which was mixed with PBS at a ratio of 3:7 (citric acid/PBS, pH 4). After washing with the same solution, 10 μL of each pepsin concentration (0, 6.25, 12.5, 25, 50, and 100 ng/mL) was dropped onto an anti-pep/GNP/PPNCs/SPCE immunosensor, respectively, allowing incubation for 2 h in a humid condition at room temperature. Because 1 mL of saliva was collected using 0.5 mL of 0.1 M citric acid for detection of pepsin in clinics [1], we used a standard solution of pepsin prepared with citric acid/PBS to mimic the real saliva sample condition. After the incubation of pepsin, the immunosensors were washed several times with citric acid/PBS.

### 2.4. Characterization and Electrochemical Measurements

The morphology of GNP/PPNCs/SPCE was investigated by SEM. CV measurements were performed to compare the electroactive behavior of the electrodes in each modification step of the immunosensor, as well as to fabricate GNP/PPNCs on SPCE. Each electrode was evaluated by CV scanning from −0.3 to 0.5 V (vs. silver pseudo-reference electrode) at a scan rate of 50 mV·s^−1^ for three cycles in 0.1 M KCl containing 2 mM [Fe(CN)_6_]^3−/4−^ as a redox indicator. The fabricated immunosensor response to various concentrations of pepsin was measured by DPV in 0.1 M KCl containing 2 mM [Fe(CN)_6_]^3−/4−^ as a redox probe, using a potential range from −0.3 to 0.5 V (vs. silver pseudo-reference electrode) at a scan rate of 10 mV s^−1^, pulse time of 20 ms, and a pulse amplitude of 100 mV. The analytical outputs were obtained from the change in DPV peak current measurements before and after the pepsin-anti-pepsin reaction (ΔI = the peak current at 0 ng/mL of pepsin—the peak current at each concentration of pepsin). All electrochemical measurements were made in triplicate, and an average was used for analysis.

### 2.5. Real Sample Test—Electrochemical Sensing of Pepsin in Saliva

The collection of saliva in LPR patients was approved by the Ethics Committee of Kyung Hee University Medical Center (KMC IRB1432-01), and all participants signed an informed consent document. LPR patients (*n* = 2) were chosen based on clinical diagnostic criteria from the Department of Otorhinolaryngology, Head and Neck Surgery. Study participants were instructed to collect saliva in the early morning before eating, drinking, or brushing their teeth, as previously reported [1]. LPR patients were given 30 mL tubes containing 0.5 mL of citric acid (0.1 M, pH 2.5) for the collection of saliva (1 mL) including sputum. Citric acid was selected as the best storage solution to preserve the activity of pepsin. Saliva samples were refrigerated at −80 °C and used for analysis within two months of collection. For analysis, saliva samples were centrifuged for 20 min at 4 °C at 14,000× *g*, and the supernatant was harvested. Pepsin concentration in saliva samples was measured using a direct enzyme-linked immunosorbent assay (ELISA). Briefly, the saliva samples (100 µL) were coated onto 96 well plates for 24 h. Each well was blocked with 5% BSA in PBS for 1 h at room temperature and washed with 1% BSA/PBS containing 0.05% Tween 20. The wells were incubated with a primary antibody (anti-pepsin antibody, 1:80) for 2 h at 37 °C. After washing, the wells were incubated with a horseradish peroxidase-coupled secondary antibody for 1 h at RT. A substrate and a stop solution were introduced sequentially. The optical density (OD450) of each well was determined within 30 min using a Synergy HT Multi-mode Microplate Reader (BioTek, Winooski, VT, USA).

Ten microliters of the same saliva samples were dropped onto the prepared electrode and kept for 2 h at room temperature. Subsequently, the DPV signal was recorded with the same procedure. Pepsin levels for three replicates were calculated using calibration equations.

## 3. Results and Discussion

### 3.1. Preparation and Characterization of GNP-Decorated PPNCs on SPCE

Nanomaterials are one of the most promising matrices designed to construct a novel and new generation electrochemical biosensor, due to their small size and efficient catalytic properties [20]. In particular, nanostructured conducting polymers, such as nanorods, nanoflowers, nanowires, and nanotubes, have emerged as promising candidates for high-performance transducer applications. The key issue with any immunosensor is reliable immobilization of a high density of antibodies with a simple immobilization process [25]. For many years, GNPs have attracted great attention as a sensing platform for electrochemical immunosensors because of their distinctive advantages, including easy preparation, high specific surface area, good biocompatibility and high electrical conductivity [26,27]. Moreover, the incorporation of GNPs shows significant amplification of the electro-ionic signals generated through binding of the antigen to its captured antibody [23]. In addition, GNPs can increase the amount of immobilized antibodies [25].

**Figure 2:**
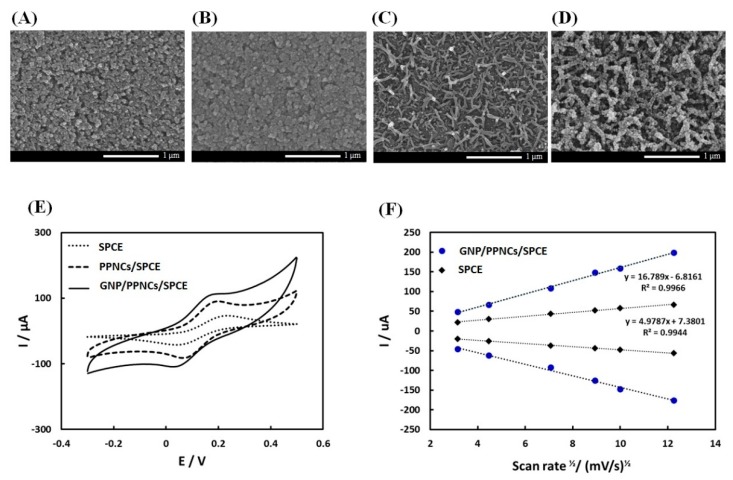
SEM images of (**A**) bare screen-printed carbon electrode (SPCE); (**B**) pre-nucleated SPCE; (**C**) NSA-doped PPNCs on SPCE (PPNCs/SPCE), and (**D**) GNP-decorated PPNC-NSA on SPCE (GNP/PPNCs/SPCE); (**E**) cyclic voltammograms of bare SPCE, PPNCs/SPCE, and GNP/PPNCs/SPCE in 0.1 M KCl containing 2 mM [Fe(CN)_6_]^3−/4−^ in the range from −0.3 to 0.5 V (vs. silver pseudo-reference electrode) at a scan rate of 50 mV·s^−1^, respectively; and (**F**) peak current versus square root of the scan rate for bare SPCE and GNP/PPNCs/SPCE, respectively.

In this study, we prepared GNP-decorated conducting PPys with coral-like structures on SPCE to improve the surface area and the antibody immobilization efficiency. The fabrication of GNP/PPNCs/SPCE proceeded via three steps. The first step was to form a thin pre-nucleation film of PPy on the surface of SPCE, which was briefly electropolymerized at 0.8 V (vs. silver pseudo-reference electrode) for 20 s in a 0.2 M KCl solution containing 0.1 M pyrrole. Figure 2A,B show the SEM images of SPCE before and after the pre-nucleation step. As shown in Figure 2B, this prenucleation film consisted of small nuclei, which served as anchors for the formation of a coral-like PPy nanostructure. The second step was to grow nanocoral PPys on pre-nucleated SPCE (Figure 2C). The growing step was performed at 0.6 V (vs. silver pseudo-reference electrode) for 120 s in a 0.01 M NSA solution containing 0.2 M pyrrole monomers to constitute micelles themselves in an aqueous solution. During the growing step, a high local electric field was formed to the edge of the small nuclei within the pre-nucleation film on SPCE, referred to as the edge effect [21]. Subsequently, these conditions caused the micelles to attach to the edge of the nuclei and grow to PPNC-NSA on SPCE. Finally, the PPNCs/SPCE was decorated by GNPs, forming GNP/PPNCs/SPCE (Figure 2D). We optimized the concentration of HAuCl_4_·3H_2_O solution (0.5 mM), based on keeping the morphology of PPNC, as shown in Figure S1. Figure 2E shows the cyclic voltammogram of bare SPCE, PPNCs/SPCE, and GNP/PPNCs/SPCE in 0.1 M KCl containing 2 mM [Fe(CN)_6_]^3−/4−^. As shown in Figure 2E, the anodic peak currents were 46.8 ± 1.0 μA in bare SPCE, 89.3 ± 2.2 μA in PPNCs/SPCE, and 119.6 ± 5.3 μA in GNP/PPNCs/SPCE, respectively. As a result, GNP/PPNCs/SPCE showed the highest anodic peak current. In addition, the influence of scan rate on the GNP/PPNCs/SPCE in the same solution was investigated. As shown in Figure 2F, the peak current was linearly proportional to the square root of the scan rate (υ^1/2^) in the range of 10–150 mV·s^−1^, indicating that this was a diffusion controlled redox process. Furthermore, the calculated electroactive surface area could be determined using the Randles-Sevcik equation for quasi-reversible electron transfer processes. From the slope of the anodic peak current versus the square root of scan rate, the electroactive surface area of GNP/PPNCs/SPCE was 3.37 times larger than that of bare SPCE. Therefore, we suggest that the excellent electrocatalytic performance of GNP/PPNCs/SPCE was attributed to the highly effective surface area.

### 3.2. Fabrication and Electrochemical Characterization of the Immunosensor

**Figure 3:**
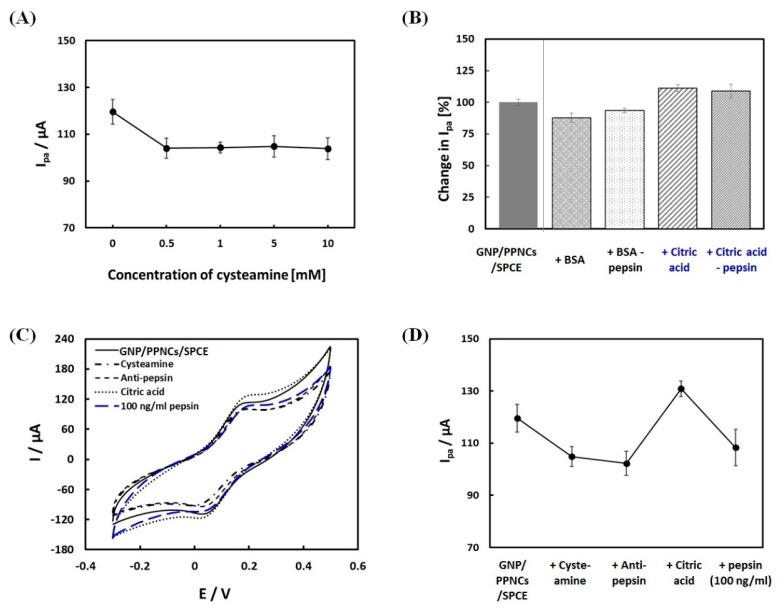
(**A**) Changes in anodic peak current (I_pa_) of the GNP/PPNCs/SPCE electrode according to cysteamine concentration; (**B**) changes in I_pa_ of GNP/PPNCs/SPCE electrode before and after the treatment of BSA and citric acid, as well as after incubation of 50 ng/mL pepsin, respectively; (**C**) electrochemical stepwise characterization of pepsin immunosensor using cyclic voltammetry (CV). CV response of GNP/PPNCs/SPCE electrode (black line), after cysteamine (mixed line), and anti-pepsin (broken line), citric acid (dotted line), and 100 mg/mL pepsin (blue broken line) treatments, respectively; and (**D**) the corresponding I_pa_ of GNP/PPNCs/SPCE electrode, after cysteamine, anti-pepsin, citric acid, and 100 mg/mL pepsin treatments, respectively.

To investigate the surface changes of the modified electrode, we performed CV measurements, which were widely used to record the changes of electrode behavior after each immobilization step, using ferricyanide as a redox indicator. At first, CA with both thiol and amine groups was used as a linker between gold and anti-pepsin to immobilize the antibody on GNP/PPNCs/SPCE. Figure 3A shows the anodic peak current (I_pa_) of CA-modified GNP/PPNCs/SPCE at different concentrations of CA (0, 0.5, 1, 5 and 10 mM). The I_pa_ decreased at 0.5 mM CA and reached a plateau with concentrations over 10 mM. The decrease of I_pa_ in GNP/PPNCs/SPCE after the modification by CA might be attributed to the self-assembly of CA monolayers, which blocked electron transfer between the GNP/PPNCs/SPCE surface and redox indicator. Though the I_pa_ values were similar in all concentrations of CA, we selected the optimal concentration as 1 mM due to the lowest observed standard deviation. After the modification of CA, anti-pepsin was immobilized on GNP/PPNCs/SPCE through the formation of an amide bond between carboxylic groups of the antibody and amine groups of CA by EDC/NHS chemistry. Any unbound anti-pepsin was removed from the GNP/PPNCs/SPCE surface by washing with a PBS solution.

For preventing non-specific binding, the anti-pep/GNP/PPNCs/SPCE electrode was incubated with citric acid/PBS (0.1 M citric acid: PBS = 3:7 volume ratio, pH 4). We utilized a citric acid/PBS solution as a blocking agent for the following reasons: (i) Citric acid was a unique capping agent to protect and stabilize the metal nanoparticles [28]. The capping effect of citric acid could effectively prevent non-specific binding of other interferences on gold surfaces. (ii) Because pepsin was activated and stabilized at low pH, under 2, human saliva samples must be collected using citric acid as a storage solution. Therefore, we prepared the standard solutions of pepsin using citric acid/PBS, similar to the clinical sample condition. (iii) As pepsin was the principal proteolytic enzyme, it might decompose the BSA which was frequently used for blocking non-specific binding. (iv) As the low pH of the pepsin solution caused a charge change in the antibody immobilized on the electrode, pretreatment of citric acid could stabilize the electrode surface before incubation with pepsin. To confirm the blocking effect of citric acid, we compared the I_pa_ of GNP/PPNCs/SPCE before and after the treatment of BSA and citric acid, as well as after incubation of 50 ng/mL pepsin, respectively. As shown in Figure 3B, the I_pa_ of GNP/PPNCs/SPCE after the BSA treatment decreased to 87.8 ± 0.8% of the initial value without any treatment. Subsequently, the incubation of pepsin caused the increase of I_pa_ in the BSA-treated GNP/PPNCs/SPCE (93.5 ± 2.6%). This increase might be attributed to the change toward positive charge of BSA in low pH. As the isoelectric point (PI) of BSA was approximately 4.4, BSA carried a net positive charge at pH values lower than PI [29]. On the other hand, the I_pa_ of GNP/PPNCs/SPCE after the citric acid treatment increased to 111.1 ± 1.5% of the initial value without any treatment. After pepsin incubation, the I_pa_ of citric acid-treated electrode maintained a similar value (108.9 ± 3.3%). These results showed that citric acid could be an effective blocking effect of the gold surface.

Figure 3C,D show the CV and I_pa_ of modified electrodes after each immobilization step, respectively. The voltammetric response of ferricyanide at the modified electrode decreased until the antibody immobilization step, because CA and antibody could prevent the electron transfer between the GNP/PPNCs/SPCE surface and ferricyanide. However, the I_pa_ increased after the treatment of citric acid/PBS, because the positively charged antibody in low pH could facilitate electron transfer of ferricyanide toward the electrode. The antibody existed as a positively charged species in citric acid/PBS (pH 4) below its PI [30]. Finally, the binding of 100 ng/mL pepsin by anti-pep/GNP/PPNCs/SPCE effectively blocked the electron transfer of ferricyanide to the electrode surface. These results showed that citric acid caused an increase of the anodic peak current, due to the electrostatic attraction between anti-pepsin and [Fe(CN)_6_]^3−/4−^ ion, as well as the effective blocking effect of the gold surface. In addition, the increase of I_pa_ after the treatment of citric acid improved the subsequent sensing performance of the immunosensor for pepsin detection, because the binding of pepsin on the electrode decreased the current. Therefore, citric acid was an effective agent for preventing non-specific binding and improving the sensing performance of anti-pep/GNP/PPNCs/SPCE for detection of salivary pepsin, as well as a pepsin storage solution.

### 3.3. Analytical Performance of the Immunosensor

**Figure 4:**
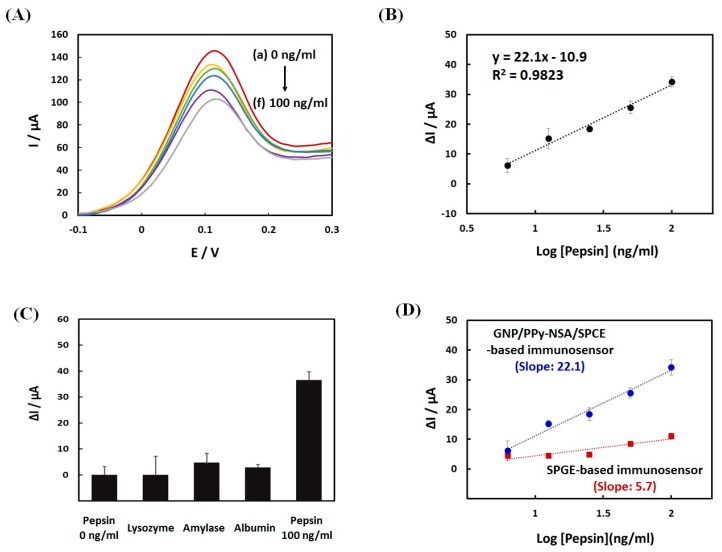
(**A**) Differential pulsed voltammetry (DPV) curves of the immunosensor after incubation with pepsin in the concentration range from 0 to 100 ng/mL; (**B**) calibration curve between the current change (ΔI) and log pepsin concentration from 6.25 to 100 ng/mL for the developed immunosensor; (**C**) changes in immunosensor current after incubation with 100 ng/mL pepsin and other interfering proteins (100 μg/mL), such as lysozyme, amylase, and albumin; and (**D**) Comparison of sensitivity toward pepsin between the GNP/PPNCs/SPCE-based immunosensor and the SPGE-based immunosensor.

The response of the immunosensor toward different pepsin concentrations was investigated by recording DPVs of 0.1 M KCl solution containing 2 mM [Fe(CN)_6_]^3−/4−^. The concentration range of pepsin from 6.25 to 100 ng/mL was selected by the previous result, which reported the average pepsin level upon waking in LPR patients as 17.2 ng/mL [1]. Figure 4A shows, the DPV curves of the immunosensor after incubation with different pepsin concentrations. The reference signal at 0 ng/mL of pepsin showed a good reproducibility with relative standard deviation (RSD) of 3.56% (*n* = 9). The resulting calibration curve was linear over the concentration range from 6.25 to 100 ng/mL (Figure 4B). The corresponding calibration regression equation was ΔI (μA) = 22.1 log [pepsin] (ng/mL) − 10.9, R^2^ = 0.9823. The limit of detection (LOD) was calculated using the standard deviation of the blank and the slope method (3.3 σ/slope), according to the ICH Q2B guidelines [31]. As a result, the LOD of this immunosensor for the lower concentration range was 2.2 ng/mL pepsin.

To examine the specificity of this immunosensor, we compared the DPV response of the immunosensor after incubation of 100 ng/mL pepsin with that after reaction with other interfering proteins, such as lysozyme (100 μg/mL), amylase (100 μg/mL), and human serum albumin (100 μg/mL), which could exist in saliva. As shown in Figure 4C, only pepsin caused a dramatic change in current, which meant that our immunosensor had a high specificity for pepsin detection.

To evaluate the reproducibility of the proposed immunosensor, a series of six electrodes were prepared for the detection of 50  ng/mL pepsin. The RSD of this immunosensor was 4.78%, suggesting the reproducibility of the immunosensor was satisfying. In addition, we performed a stability test of this immunosensor at 4 °C. After a storage period of two weeks, the immunosensor retained 91.6% of its initial reference response. Therefore, we suggest that this immunosensor had good stability.

We compared the sensitivity of the GNP/PPNCs/SPCE-based immunosensor with that of an SPGE-based immunosensor, which was prepared by the same fabrication process. As shown in Figure 4D, the sensitivity of the GNP/PPNCs/SPCE-based immunosensor was 3.9 times higher than that of the SPGE-based immunosensor in the concentration range from 6.25 to 100 ng/mL. The improved sensing performance of our immunosensor might be attributed to the large surface area of GNP/PPNCs on SPCE. In addition, the performance of our immunosensor was compared to that of other pepsin detection methods in Table 1.

### 3.4. Real Sample Analysis

Saliva is a difficult matrix to manage for biological analysis, because many components in saliva can affect the response of the analyte of interest. In particular, immunosensors are subject to matrix effects, because the interaction between the antigen (analyte) and antibody is hindered due to matrix interference [34]. However, appropriate sample preparation procedures, including dilution, centrifugation, filtration, precipitation and extraction, can help eliminate or minimize matrix effects [35]. For example, freezing/centrifugation treatment was very effective to minimize the clogging effect of highly viscous mucins in saliva [36]. Therefore, saliva samples in this study went through 1.5 times dilution with citric acid, freezing and centrifugation to reduce matrix effects. To evaluate the preliminary feasibility of our immunosensor, it was utilized to quantify pepsin concentrations in saliva of LPR patients (*n* = 2). Each sample was measured three times, using three freshly prepared electrodes. The obtained results were compared with those of a direct ELISA as a reference method. As shown in Table 2, the results showed good agreement between the two methods with a relative error of 12.0%. Therefore, the fabricated GNP/PPNCs/SPCE-based immunosensor might be applied as a useful tool for the quantification of salivary pepsin levels.

## 4. Conclusions

In summary, a facile and disposable electrochemical immunosensor for pepsin detection in saliva was developed using GNP-decorated PPNCs on SPCE. The GNP/PPNCs/SPCE could provide a large number of active sites for immobilizing antibodies and promote the electron transfer to the electrode surface. As a result, the sensitivity of the GNP/PPNCs/SPCE electrode for pepsin was 3.9 times higher than that of the bare SPGE. As our immunosensor possessed good sensitivity and high specificity for pepsin detection under acidic condition, we successfully measured the pepsin levels in real saliva samples of LPR patients. Moreover, the results agreed with those of the conventional ELISA method. Therefore, we expect that this immunosensor for pepsin detection under acidic condition would be a useful tool for preliminary LPR diagnosis.

## Figures and Tables

**Table 1:** Sensing performance comparison of the developed immunosensor with other pepsin detection methods.

Detection Method	Linear Range (ng/mL)	Lower Detection Limit (ng/mL)	Ref.
ELISA (O.D. 450 nm)	0.1–10	0.1	[23]
Enzymatic assay with a fluorescent substrate	12.5–400	-	[24]
Colorimetric analysis—LFD ^1^	-	16	[32]
Fluorescence sensor based on AuNCs@Lyz ^2^	1000–100,000	256	[33]
Electrochemical immunosensor	6.25–100	2.2	In this work

^1^ Lateral flow device, Peptest^TM^ (RD Biomed Ltd.); ^2^ Lysozyme-stabilized Au nanoclusters.

**Table 2:** Pepsin levels in patient saliva samples (*n* = 2) obtained by the as-prepared immunosensor and the ELISA method.

Human Saliva Samples No.	Proposed Method (ng/mL)	ELISA Method (ng/mL)	Relative Error (%)
1	4.3	4.0	7.5
2	10.3	9.2	12.0

## Supplementary Materials

The following are available online at http://www.mdpi.com/1424-8220/18/6/1685/s1, Figure S1: The SEM images of GNPs/PPNCs/SPCE obtained from (A) 0, (B) 0.5, (C) 1, and (D) 5 mM HAuCl_4_·3H_2_O solutions containing 0.1 M KCl.
