# Supplementary material for: Label-Free Detection of Salivary Pepsin Using Gold Nanoparticle/Polypyrrole Nanocoral Modified Screen-Printed Electrode
Source: Sensors (Basel). 2018 May 24;18(6):1685. doi: 10.3390/s18061685 (PMC6021850; doi:10.3390/s18061685)
Supplement: Supplementary file 1 [file sensors-18-01685-s001.pdf]

Supporting Information

Label-free detection of salivary pepsin using gold nanoparticle / polypyrrole nanocoral modified screen-printed electrode

Doyeon Lee[1], Young Ju Lee[2], Young-Gyu Eun[3], and Gi-Ja Lee[1,2,*]

[1] Department of Medical Engineering, Graduate School, Kyung Hee University, Seoul 130-701, Korea

[2] Department of Biomedical Engineering, College of Medicine, Kyung Hee University, Seoul 130-701, Korea

[3] Department of Otolaryngology–Head and Neck Surgery, Kyung Hee University Medical Center, Seoul 130-702, Korea

* To whom correspondence should be addressed. E-mail: gjlee@khu.ac.kr

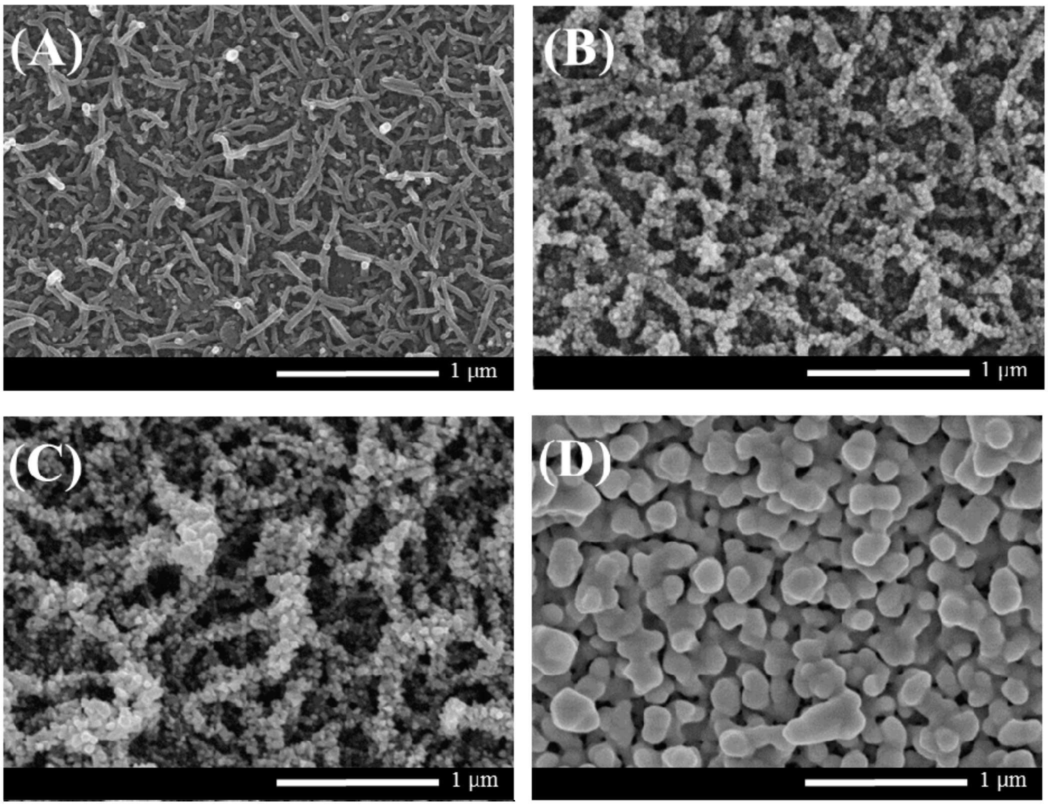

Figure S1. The SEM images of GNPs/PPNCs/SPCE obtained from (A) 0, (B) 0.5, (C) 1, and (D) 5 mM $HAuCl_4 \cdot 3H_2O$ solutions containing 0.1 M KCl.
